# A Deliberate Suppression of the Facts

**Published:** 1919-04-05

**Authors:** Chas. A. Mercier


### THE UNIFORM SYSTEM OF ACCOUNTS.

### A Deliberate Suppression of the Facts.

By CHAS. A. MERCIER, M.D., F.R.C.P., F.R.C.S.

Mr. Thies, formerly Secretary of the Royal Free Hospital, is an ingenious man. He has a remarkable power of omission. In a long address that he has recently delivered to the Association of Hospital Officers, he made mention of the Uniform System of Accounts; and, if the *Hospital Gazette* is to be believed, he contrived to speak at some length on the origin and history of this system without making any mention whatever of its author and originator, Sir Henry Burdett. This is more than describing the play of Hamlet without mentioning the Prince of Denmark. It is describing the Peninsular War without mention of Wellington. It is describing the invasion of Russia in 1812 without mentioning Napoleon. It is, therefore, a feat of considerable ingenuity. Mr. Thies dates the devising of the Uniform System of Accounts from a meeting of the Hospital Sunday Fund in 1891, an error of only 22 years, since the scheme was actually devised by Sir Henry Burdett, with the assistance of Mr. Laundy, in 1869. What was done by the Hospital Sunday Fund in 1891 was to adopt, or rather, to recommend the hospitals concerned to adopt, a system that had long been in use and had been found very successful. It was devised for the Queen's Hospital at Birmingham, it was in use in London sixteen years before the meeting of the Metropolitan Hospital Sunday Fund recommended its adoption. In 1903 the Secretaries of King Edward's Hospital Fund added their voices to the chorus of approval, and declared that to pretend that the true position of any institution can be shown without such a balance sheet as was advised by Sir Henry Burdett would be an injustice to the Uniform System of Accounts. The then Prince of Wales, his present Majesty, in a speech reported in the *Times* on Christmas Day 1908, insisted on the value of the System in enforcing control over expenditure, and the System has so great an influence upon the whole administration of the institutions in which it is adopted, and is adopted so generally, that it may truthfully be said that the history of the Uniform System of Accounts is the history of voluntary hospitals in this country.

Nor is it in this country alone, nor in hospitals alone, that the Uniform System of Accounts is adopted. Its advantages are so great and so manifest that it is in use throughout the Empire. It has been adopted by the Governments of Victoria, Queensland, New Zealand, and of several Crown Colonies. It is enforced in the principal hospitals in New South Wales, South Australia, Western Australia, South Africa and Tasmania. It is employed by one of the principal hospitals in the United States of America, and all the war hospitals run by the British Red Cross Society and the Order of St. John of Jerusalem, and has spread to charitable and other institutions of all kinds.

The Uniform System of Accounts has, in short, been of incalculable benefit to hospitals and voluntary institutions of all kinds. It owes its origin entirely, and its present shape almost entirely, to the practical genius of Sir Henry Burdett, and is as often called Burdett's System of Accounts as by the title that he gave to it; and to omit all mention of his name in giving what purports to be a history of the System shows either a strange ignorance or a still stranger animus.

